# Notes on Michael Schülke’s pselaphine collections from China. – Tyrini. II. Genus *Megatyrus* Hlaváč & Nomura (Coleoptera, Staphylinidae, Pselaphinae)

**DOI:** 10.3897/zookeys.301.4912

**Published:** 2013-05-17

**Authors:** Zi-Wei Yin, Li-Zhen Li

**Affiliations:** 1 Department of Biology, College of Life and Environmental Sciences, Shanghai Normal University, 100 Guilin Road, Shanghai, 200234, P. R. China

**Keywords:** Staphylinidae, Pselaphinae, taxonomy, *Megatyrus*, new species, China

## Abstract

Two new species of the tyrine genus *Megatyrus* Hlaváč & Nomura, *Megatyrus schuelkei* Yin & Li, **sp. n.** (based on two males) and *Megatyrus tengchongensis* Yin & Li, **sp. n.** (based on one female), from Yunnan, Southwest China are described, illustrated and distinguished from allied species. The body size, form of maxillary palpi, male and female genital structures, and distributional patterns are used to separate the new species.

## Introduction

Members of *Megatyrus* Hlaváč & Nomura (type species: *Megatyrus menglianensis* Hlaváč & Nomura) are large (3.30–4.12 mm), rare pselaphine rove beetles inhabiting leaf litter of the forest floor. Six species have been described from South Asia: one from China, two from Vietnam (Hlaváč and Nomura 2003), and three recently described (Nomura et al. 2011) from Thailand. *Megatyrus* is morphologically similar to the genera *Tyrus* Aubé and *Tyrodes* Raffray in sharing a similar general habitus and pronotal structure. It can be readily separated from both by the much larger body size, the more pedunculate maxillary palpi, the absence of a palpal cone, and the abdominal tergite IV (first visible tergite) being clearly longer than tergite V. A recent study on the pselaphine collections of Michael Schülke, all collected during several expeditions to China, produced numerous undescribed species. After a comparative study on the types of all known species of *Megatyrus* (all in National Museum of Nature and Science, Tokyo, Japan [NSMT]), the present study reports two new species, as the second of a series dealing with Schülke’s material. Diagnoses, descriptions, and illustrations of major diagnostic features of the new species are provided.

## Material and methods

The material treated in this study is housed in the following public institution and private collection:

**SNUC** Insect Collection of Shanghai Normal University, Shanghai, China (Zi-Wei Yin)

**pcMS** private collection of Michael Schülke, Berlin, German

The collection data of the referred material are quoted verbatim. A slash (/) is used to separate lines on the same label, and a double slash (//) is used to separate different labels. Authors’ notes are included in a ‘[]’. All type material bears the following label: ‘HOLOTYPE [red] or PARATYPE [yellow] / [genus name, species name] / sp. n., [authors of the species] / det. 2013. The depository is indicated after the collection data of the respective species.

All measurements are in millimeters. The following acronyms are applied: **AL**–length of the abdomen along the midline; **AW**–maximum width of the abdomen; **BL**–length of the body (= HL + PL + EL + AL); **EL**–length of the elytra along the sutural line; **EW**–maximum width of the elytra; **HL**–length of the head from the anterior clypeal margin to the occipital constriction; **HW**–width of the head across eyes; **PL**–length of the pronotum along the midline; **PW**–maximum width of the pronotum.

### Descriptions of new species

#### Megatyrus schuelkei

Yin & Li sp. n.

urn:lsid:zoobank.org:act:FCA38920-031F-4F09-8060-A455CB8927B1

http://species-id.net/wiki/Megatyrus_schuelkei

Figs 1A , 2

##### Type material

(2 ♂♂). Holotype: ♂, labeled ‘CHINA: Yunnan, Lincang Pref., / Xue Shan, 48 km N Lincang, / 2070 m, 24°19'03"N, 100°07'13"E, / forest remnant, N-slope, litter & / mushrooms sifted, 12.IX.2009, / leg. M. Schülke [CH09-45]’ (pcMS). Paratype: 1 ♂, same label data as holotype (SNUC).

##### Description.

**Figure 1.**
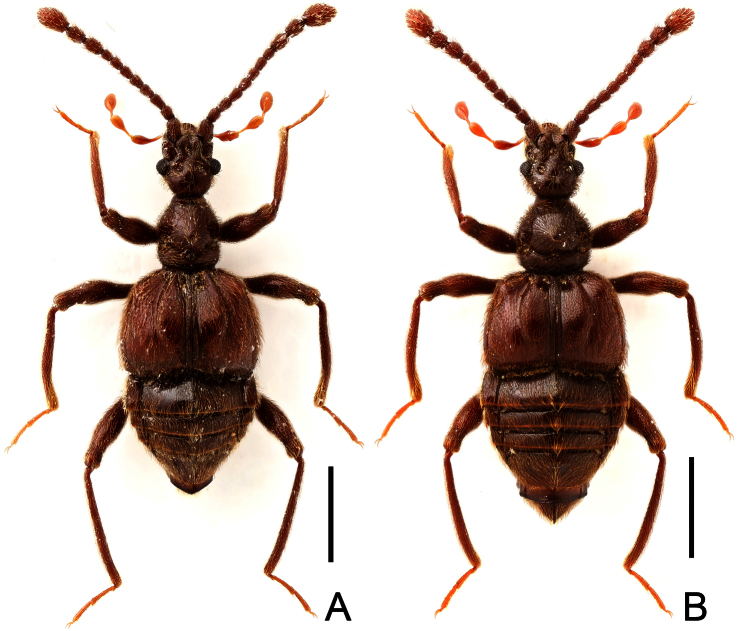
Habitus of *Megatyrus*. **A** *Megatyrus schuelkei*, male **B** *Megatyrus tengchongensis*, female. Scales (mm): 1.0.

**Figure 2.**
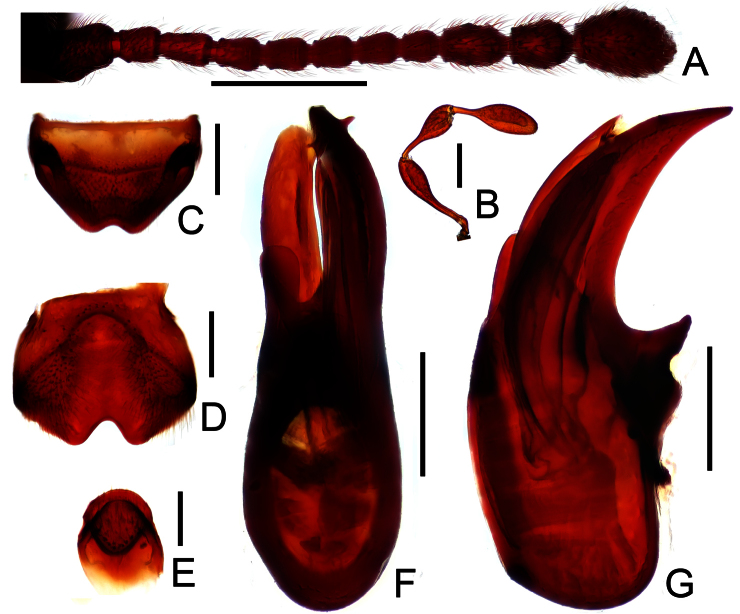
Diagnostic features of male *Megatyrus schuelkei*. **A** antenna **B** maxillary palpus **C** tergite VIII **D** sternite VIII **E** sternite IX **F** aedeagus, in dorsal view **G** same, in lateral view. Scales (mm): **A** = 0.5; **B, C, D, F, G** = 0.2; **E** = 0.1.

Male (Fig. 1A). Length 3.52–3.70 mm. Head longer than wide, HL 0.81–0.84 mm, HW 0.68–0.69 mm; eyes each composed of about 50 facets; maxillary palpi as in Fig. 2B; antennae (Fig. 2A) with scapes roundly projecting basolaterally; terminal three antennomeres enlarged. Pronotum longer than wide, PL 0.71–0.73 mm, PW 0.64–0.65 mm, lateral margins nearly parallel, evenly narrowed apically at basal 2/3. Elytra wider than long, EL 0.97–0.99 mm, EW 1.43–1.48 mm. Mesotrochanters, metatrochanters and metafemora lacking spine or projection at ventral margins. Abdomen broad at base and narrowed apically, AL 1.03–1.14 mm, AW 1.38–1.40 mm. Tergite VIII as in Fig. 2C; sternite VIII as in Fig. 2D; sternite IX as in 2E. Aedeagus length 0.83 mm, with elongate median lobe asymmetric (Figs 2F, G).

Female. Unknown.

##### Comparative notes.

*Megatyrus schuelkei* is most similar to *Megatyrus laqueus* Hlaváč & Nomura from Vietnam in sharing similar body size and aedeagal structure. The two species can be clearly separated by the more elongate maxillary palpi, and the antennomeres VI–VIII are slightly more elongate in the new species. *Megatyrus laqueus* has shorter maxillary palpi and quadrate antennomeres VI–VIII. *Megatyrus schuelkei* can be separated from *Megatyrus menglianensis* by the more elongate pronotum, and different aedeagal structure.

##### Distribution.

Southwest China: Yunnan.

##### Biology.

Individuals were collected from sifted leaf litter with mushrooms in a forest remnant.

##### Etymology.

The new species is named after Michael Schülke, collector of the type series.

#### Megatyrus tengchongensis

Yin & Li sp. n.

urn:lsid:zoobank.org:act:989B0696-4F01-47B8-9279-DAE226D8D119

http://species-id.net/wiki/Megatyrus_tengchongensis

Figs 1B , 3

##### Type material

(1 ♀). Holotype: ♀, labeled ‘CHINA: Yunnan [CH07-17] / Baoshan Pref., mountain range 25 km S / Tengchong, 1900 m, 24°48'28"N, 98°32'03"E, dev. primary decid. forest, / litter, fungi sifted, 2.V.2007, M. Schülke’ (pcMS).

##### Description.

**Figure 3.**
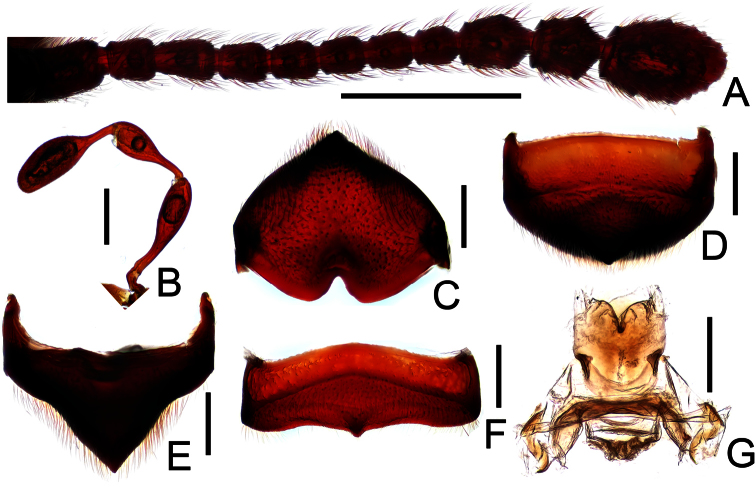
Diagnostic features of female *Megatyrus tengchongensis*. **A** antenna **B** maxillary palpus **C** tergite VIII, in posterior view **D** same, in dorso-posterior view **E** same, in dorsal view **F** sternite VIII **G** female genital complex, in dorsal view. Scales (mm): **A** = 0.5; **B–G** = 0.2.

Female (Fig. 1B). Length 3.71 mm. Head longer than wide, HL 0.79 mm, HW 0.65 mm; eyes each composed of about 35 facets; maxillary palpi as in Fig. 3B; antennae (Fig. 3A) with scapes simple; terminal three antennomeres enlarged. Pronotum slightly longer than wide, PL 0.74 mm, PW 0.71 mm, lateral margins nearly parallel, evenly narrowed apically at middle. Elytra wider than long, EL 0.93 mm, EW 1.43 mm. Legs simple. Abdomen broad at base and narrowed apically, AL 1.25 mm, AW 1.46 mm. Tergite VIII as in Fig. 3C–E; sternite VIII as in Fig. 3F. Genital complex weakly sclerotized, width 0.55 mm, with dorsal and ventral sclerites (Figs 3G).

Male. Unknown.

##### Comparative notes.

In general, *Megatyrus* males are morphologically similar to females, and possess indistinct second sexual characters. Proportions of head, pronotum and abdomen between male and female are close, except that females have much shorter elytra, as well illustrated in Nomura et al. (2011: 123). The single female of *Megatyrus tengchongensis* is very similar to that of *Megatyrus menglianensis* Hlaváč & Nomura by the tergite VIII possessing a large and thick median projection, but the two species can be separated by the maxillary palpomeres I being more elongate in *Megatyrus tengchongensis*, and the clearly different structure of the genital complex. From *Megatyrus schuelkei* described above, *Megatyrus tengchongensis* can be separated by the relatively much shorter and stouter pronotum.

##### Distribution.

Southwest China: Yunnan.

##### Biology.

The adult was collected from sifted leaf litter in a deciduous forest.

##### Etymology.

The new species is named after the type locality, Tengchong County.

## Supplementary Material

XML Treatment for Megatyrus schuelkei

XML Treatment for Megatyrus tengchongensis
